# Strengthening recovery: the impact of paired exercises in geriatric hip fracture rehabilitation—the PaLMe project

**DOI:** 10.1007/s41999-025-01192-1

**Published:** 2025-04-02

**Authors:** Eyal Yaacobi, Shanny Gur, Adi Berliner Senderey, Ben Kapner, Tom Mushkat, Gili Golan, Smadar Pasand Van-Stee, Tova Farjun, Nissim Ohana

**Affiliations:** 1 Department of Orthopaedic Surgery, Meir Medical Center Affiliated with the Faculty of Health Sciences and Medicine, Tel Aviv University, 59 Tchernichovsky St., kfar-Saba, Israel; 2 Innovation Division, Clalit Research Institute, Clalit Health Services, Ramat Gan, Israel; 3 Department of Biomedical Informatics, Harvard Medical School, Boston, MA USA; 4 The Ivan and Francesca Berkowitz Family Living Laboratory Collaboration at Harvard Medical School and Clalit Research Institute, Boston, MA USA; 5 Computer Science Department, Reichman University, Herzliya, Israel; 6 Department of Psychology, Faculty of Social Sciences, The Hebrew University of Jerusalem, Jerusalem, Israel

**Keywords:** Old population rehabilitation, Hip fracture recovery, Paired exercises, Online rehabilitation, Functional outcomes, Psychological well-being

## Abstract

**Purpose:**

This study aimed to evaluate the effectiveness of the PaLMe concept, which incorporates paired exercises into rehabilitation for old patients recovering from hip fracture surgery, and to assess the feasibility of implementing an online rehabilitation program in this population.

**Methods:**

A prospective, randomized interventional study was conducted in a hospital setting. A total of 93 participants (mean age: 80.5 years, 66% women) were enrolled and randomized into an intervention group (PaLMe, paired exercises, n = 44) or a control group (individual exercises, n = 49). The study intervention lasted for the duration of the hospital stay, with an average length of 5.6 days. In the PaLMe group, participants were paired using a custom algorithm developed to match patients based on similar clinical and demographic characteristics, fostering interaction and mutual motivation during synchronized exercises. Walking ability was measured by distance walked and level of assistance required, while self-efficacy and motivation were assessed using validated scales. Feasibility was evaluated through recruitment and retention rates, adherence to prescribed sessions, and participant feedback on usability and satisfaction. Data were analyzed using independent t-tests and qualitative feedback.

**Results:**

The PaLMe group demonstrated significantly greater improvements in walking distance, with 66% achieving ≥ 21 m at discharge compared to 43% in the control group (*p* < .05). Self-efficacy and motivation also improved more in the PaLMe group. Feasibility metrics indicated high program acceptability, with recruitment and retention rates of 37% and 89%, respectively. Adherence to the program was moderate, with 47% of PaLMe participants completing more than 50% of prescribed sessions. Participant feedback highlighted increased confidence, motivation, and satisfaction with the paired exercise format, despite logistical challenges such as scheduling conflicts.

**Conclusion:**

The PaLMe program significantly enhances physical and psychological recovery in senior patients after hip fracture surgery, demonstrating its potential as an effective rehabilitation strategy. Feasibility findings indicate strong acceptability but highlight logistical barriers, such as scheduling conflicts and adherence challenges, which must be addressed to optimize implementation. Further research is warranted to explore long-term outcomes and scalability across broader settings.

## Key summary points

**Aim:**

This study investigates whether paired exercises in an online rehabilitation program improve physical and psychological recovery in older adults after hip fracture surgery.

**Findings:**

Paired exercises significantly improved walking distance, self-efficacy, and motivation compared to individual exercises, with 66% of the intervention group achieving walking distances of ≥ 21 m. Feasibility metrics showed high retention rates but moderate adherence due to logistical challenges.

**Message:**

Paired exercises in rehabilitation programs can enhance recovery outcomes and offer a scalable, effective approach for older adults recovering from hip fractures.

## Introduction

Hip fractures, predominantly affecting the old population, pose a significant public health challenge due to their association with severe physical impairment, prolonged disability, and elevated mortality rates [1–4]. Mortality within the first year can range from 20 to 35%, highlighting the seriousness of these injuries [5–7]. Effective management requires prompt surgical stabilization, early mobilization, and comprehensive rehabilitation, encompassing physical therapy, psychological support, and social reintegration to address both physical and mental recovery [8]. Without integrated care, patients face declines in mobility, increased dependence, and reduced quality of life [3–9], underscoring the importance of a long-term, multidimensional approach to maximize recovery and outcomes.

Rehabilitation plays a critical role in achieving optimal outcomes following hip fractures in older people, yet adherence to these regimens can be challenging [10]. Supervised physical therapy is significantly more effective than home-based or unsupervised exercises, particularly in old patients [11, 12]. For instance, Latham et al. [13], found substantial improvements in physical performance after six months of supervised exercise in patients recovering from hip fractures, compared to those in home-based programs. Despite these clear benefits, many patients struggle with adherence due to barriers like pain, lack of motivation, and feelings of social isolation [11, 14]. Novel interventions are needed to enhance patient engagement and promote sustained participation in rehabilitation [15, 16].

Psychosocial factors, including mental well-being, social support, and self-efficacy (defined as an individual’s belief in their ability to successfully execute behaviors necessary to achieve a specific goal), also play a critical role in recovery [15, 17]. Patients undergoing surgery for hip fractures often experience declines in mental health, including symptoms of depression, anxiety, and hopelessness [18–21]. These challenges can exacerbate postoperative pain and hinder recovery by reducing motivation to engage in rehabilitation [22]. Social support, especially through peer networks, has been linked to better mental health outcomes, improved rehabilitation adherence, and enhanced physical recovery [23]. Incorporating mental health and social support strategies into rehabilitation program is therefore crucial for improving long-term outcomes [24, 25].

Unlike traditional models where patients undergo rehabilitation independently between supervised physiotherapy sessions [11], shared settings have been shown to address challenges such as isolation and low motivation [26]. The PaLMe (**Pa**tient **L**ike **Me**) initiative was developed as an experimental tool to evaluate whether independent, paired exercises could enhance adherence and recovery outcomes during unsupervised periods. The program improved adherence, self-efficacy, and recovery outcomes, particularly functional mobility (e.g., walking distance and balance), psychological resilience (e.g., reduced anxiety and depression), and increased engagement in rehabilitation [27], with social support playing a key role in fostering participation, motivation, and positive expectations., with social support playing a key role in fostering participation, motivation, and positive expectations [28]. Group-based activities also improve long-term adherence across various health challenges, including chronic conditions and rehabilitation settings [29–31]. Building on this evidence, the PaLMe program pairs patients recovering from femoral neck fracture surgery with others sharing similar clinical and demographic characteristics [32] Before this trial, a pilot study was conducted to test the feasibility of the platform and refine its pairing algorithm. Insights from the study were used to design the current trial.

As digital health solutions advance, artificial intelligence (AI) is increasingly utilized in rehabilitation programs to optimize patient engagement and personalize interventions. However, AI-driven models rely on real-world clinical validation to ensure their applicability in patient care. This study aimed to evaluate the effectiveness of the PaLMe concept in enhancing rehabilitation outcomes and to assess the feasibility of implementing an online rehabilitation program for older patients recovering from hip fracture surgery.

## Methods

### Study design

This was a prospective, randomized controlled trial conducted at a single institution. The study received approval from the Institutional Review Board and adhered to the principles of the Helsinki Declaration. The trial was designed and reported following the CONSORT guidelines for randomized controlled trials [33].

#### Ethics statement

The study protocol was reviewed and approved by the Institutional Review Board (IRB) of Meir Medical Center [MMC-0256-22]. All procedures performed in this study involving human participants were conducted in accordance with the ethical standards of the Helsinki Declaration.

#### Informed consent statement

Written informed consent was obtained from all participants prior to their enrollment in the study. Participants were informed about the study’s objectives, procedures, potential risks, and benefits before signing the consent form. They were assured that participation was voluntary and that they could withdraw from the study at any time without consequences for their medical care.

### Participants

#### Eligibility criteria

Participants ≥ 65 years who were admitted for hip fracture, who had **all** the following:

1. Scheduled for surgery within 48 h of admission.
2. Medically and cognitively capable of commencing rehabilitation immediately after surgery. Cognitive function was assessed based on clinical evaluation by the attending medical team, with additional bedside cognitive screening (e.g., Mini-Cog) performed in cases of uncertainty. Caregiver reports on pre-fracture cognitive function were also considered in the assessment.
3. Admitted Saturday through Wednesday

Participants were excluded if they had **one** of the following:

1. Medically ineligibility for surgery within 48 h.
2. Inability to provide informed consent.
3. Unable to follow instructions due to severe medical disabilities or psychogenic conditions.

Patients admitted on Thursdays or Fridays were excluded due to the reduced availability of rehabilitation staff over the weekend, which could delay the initiation of postoperative physical therapy—a key factor in optimizing recovery.

##### Recruitment process

Recruitment began consecutively in the orthopedic ward in November 2023, with written informed consent obtained from all participants.

##### Randomization and allocation

Participants were randomized into the intervention group (PaLMe) or the control group (individual exercises) using a two-step process to ensure balanced allocation. First, a computer-generated randomization sequence was used to stratify participants by gender and admission date. Second, for participants allocated to the PaLMe group, a custom pairing algorithm—developed specifically for this study—was employed. This algorithm matched patients based on similar clinical and demographic characteristics, such as age, gender, baseline health status, and postoperative condition (including weight-bearing status, pain levels, presence of complications, and early mobility status following surgery), to optimize compatibility and foster interaction during synchronized online exercises.

Upon enrollment, participants provided informed consent and completed a preoperative hobbies questionnaire via the Research Electronic Data Capture (Redcap) system, a secure web-based platform designed for data collection and management. Redcap, developed by Vanderbilt University, is widely used in academic and clinical research to ensure the accuracy, security, and efficiency of data management.

Following surgery, all participants were asked to perform exercises alongside asynchronous demonstrations on a screen by their bedside. Participants in the intervention group performed synchronized exercises with their matched partners during online physical therapy sessions, while those in the control group completed the exercises individually.

The randomization and allocation process were conducted by an independent researcher not involved in recruitment or data collection. Allocation concealment was maintained using sealed, opaque envelopes, which were opened only after participant enrollment was finalized.

##### Intervention

*PaLMe group* Participants in the intervention group were paired using the PaLMe platform, which matches patients based on clinical and demographic characteristics, such as age, gender, hospitalization duration and health status. Since most participants shared common comorbidities (e.g., diabetes mellitus, hypertension), clinical matching was performed based on medical file review, focusing on functional status, mobility levels, and cognitive function. Prior to the study, the department's physical therapy (PT) team filmed video clips demonstrating the exercises. These video clips were used throughout the study, with patients accessing them daily via iPads provided to each participant. Paired participants engaged in synchronized physical therapy sessions from their hospital rooms, interacting with their partners in real time while watching the instructional videos and communicating with each other through the platform. The exercises were designed to improve mobility, strength, and flexibility and included seated and bed-based movements. Sessions were scheduled three times daily: one seated morning session and two bed-based afternoon sessions, each lasting 15 min.

*Control group* Participants in the control group performed the same exercises independently, using asynchronous instructional videos delivered via bedside tablets. Both groups followed an identical rehabilitation protocol designed to promote early mobilization, with the same exercises, duration, and frequency. The only difference was that the PaLMe group performed the exercises in a paired format, while the control group completed them individually.

The primary endpoint, walking distance, was pre-specified during the study design and dichotomized into two categories: ≥ 21 m (considered clinically significant functional recovery) and < 21 m (indicating lesser recovery). Walking distances were measured over three consecutive days during hospitalization using standardized protocols. Measurements were performed at consistent times of the day by physiotherapists to ensure uniformity.

##### Pilot study

Before the main trial, a proof-of-concept pilot study was conducted to evaluate the feasibility of the PaLMe platform and identify potential technical and logistical challenges. The pilot study focused on testing key components, such as the video clips, iPad usability, patient cooperation, and any barriers to implementation, including scheduling or adherence issues. Insights gained from the pilot study were used to refine the pairing algorithm and optimize the intervention for the main trial.

### Outcomes

#### Primary outcomes

1. Walking ability was assessed using two complementary measures: the distance participants could walk and the level of assistance required during walking.
  - Walking Distance: Measured by physiotherapists over three consecutive days during hospitalization using standardized procedures. Distances were categorized as “ < 6 m,” “6–10 m,” “11–20 m,” or “ ≥ 21 m.”
  - Assistance Levels: Evaluated through self-reports and physiotherapist assessments. Assistance levels were categorized as “Independent (no assistance),” “Assistance from two people,” “Assistance from one person,” or “Supervision only (no physical assistance).”
2. Feasibility: was evaluated using multiple metrics, including recruitment and retention rates, adherence to prescribed sessions, participant satisfaction (as measured through qualitative feedback), and technical challenges (such as device issues or scheduling conflicts). These metrics were systematically recorded throughout the study.
3. Complications: Assessed included pressure sores and venous thrombosis, as these are two of the most common and preventable complications influenced by early mobilization after hip fracture surgery. Other complications were monitored but not predefined as primary outcomes since they are less directly related to rehabilitation efficacy.

#### Secondary outcomes

1. Subjective Measures using standardized tools including the EQ5D Quality of Life Questionnaire [34] and PROMIS-10 [35]. Participants self-reported pain (scale of 1–10), perceived physical health, self-efficacy (defined as confidence in their ability to engage in and complete rehabilitation exercises), and mood (each on a scale of 1–5). Task ease was assessed through participant feedback using a 5-point Likert scale, where patients rated the perceived difficulty of the exercises from ‘very easy’ to ‘very difficult. Motor abilities were evaluated using standardized physiotherapy assessments, including the ability to maintain balance during standing exercises, lower limb strength tests, and coordination during guided movement patterns
2. Adherence: Percentage of prescribed exercises completed during the study period.

Participants who completed less than 50% of the prescribed exercises were excluded from the final analysis. This decision was pre-specified during the study design to ensure data quality and meaningful interpretation of the intervention's effectiveness. This threshold was selected to focus on participants who engaged substantially with the program, allowing a more accurate assessment of its impact. Details of this criterion were outlined in the study protocol prior to data collection.

### Exercise supervision

All participants were instructed by physiotherapists on proper exercise techniques before starting the rehabilitation program. While the PaLMe group benefited from peer motivation and guidance during sessions, the nursing team monitored all participants to ensure safety and adherence. Participants completed daily self-assessments on exercise difficulty and perceived rehabilitation progress.

### Data collection

Baseline demographic and clinical data were collected on enrollment. Walking distances and complications were recorded throughout the hospital stay, while subjective outcomes were assessed at discharge using standardized tools. All data were securely managed using the REDCap platform. Following randomization, only participants who completed at least 50% of the prescribed exercises were included in the final analysis.

### Sample size

A power analysis was conducted to determine the minimum number of participants needed to detect a clinically meaningful difference between the intervention and control groups [36]. The power calculation was based on detecting a clinically meaningful difference in the proportion of participants achieving the primary endpoint (≥ 21 m walking distance at discharge). We estimated a 10% difference between the PaLMe and control groups, with an expected incidence of the endpoint in 50% of the control group based on prior rehabilitation studies. Using a two-tailed test with α = 0.05 and power = 0.8, a minimum of 86 participants were required. To account for an anticipated dropout rate, 93 participants were enrolled. However, we acknowledge that dichotomous outcomes with low incidence rates may introduce limitations to the power calculation, and the interpretation of findings should consider this constraint.

### Statistical analysis

To assess the impact of online physical therapy on both clinical and subjective outcomes, we employed a comprehensive statistical approach. Data were sourced from two platforms, REDCAP and NESS technologies, provided as JSON files and structured into eight data tables. Descriptive statistics were generated using MS-SQL queries, with advanced statistical analysis and visualizations conducted in Python, utilizing libraries such as numpy, pandas, scipy. stats, matplotlib, and seaborn. Before selecting the appropriate statistical tests, the distribution of the data was assessed using both visual and statistical methods. Normality of continuous variables was evaluated through visual inspection of histograms and Q–Q plots. Additionally, statistical tests for normality, such as the Shapiro–Wilk test, were employed to confirm the distribution of the data. Given that the data met the assumptions of normality, parametric tests were deemed appropriate for the analysis of continuous variables. Where data were not normally distributed, appropriate non-parametric tests were applied. Logistic regression models were used to estimate odds ratios (OR) for primary clinical outcomes, such as pressure sores and venous thrombosis. The key independent variable was the experimental condition (group vs. individual physical therapy), and sociodemographic factors (age, gender, health provider membership, etc.) were included to control for confounding effects. For subjective outcomes (e.g., pain, physical health, self-efficacy, mood), strong inter-variable correlations were aggregated, while weaker correlations were analyzed individually. Mixed-model analysis accounted for repeated measures across participants.

## Results

The study was conducted over an eight-month period, with the intervention phase running from November 1, 2023, to June 1, 2024. Data extraction occurred between June 2 and August 2, 2024, followed by the final analysis.

### Participant flow

A total of 250 patients were assessed for eligibility. Of these, 117 patients (47%) were excluded for not meeting the inclusion criteria, primarily due to pre-existing conditions (such as severe cognitive impairment, significant neurological disorders affecting mobility, medical instability, or musculoskeletal conditions that limited participation in rehabilitation), inability to provide consent, or cognitive impairments. An additional 64 patients (25.5%) were excluded for medical reasons (including acute delirium, respiratory or cardiovascular complications, or severe uncontrolled postoperative pain that impaired participation in rehabilitation), while 52 patients (20.7%) were excluded due to technical issues, such as device incompatibility or difficulty accessing the digital platform. Only 17 patients (6.8%) refused to participate. A total of 93 participants were enrolled and randomized into the intervention (PaLMe) and control groups.

**Fig. 1:**
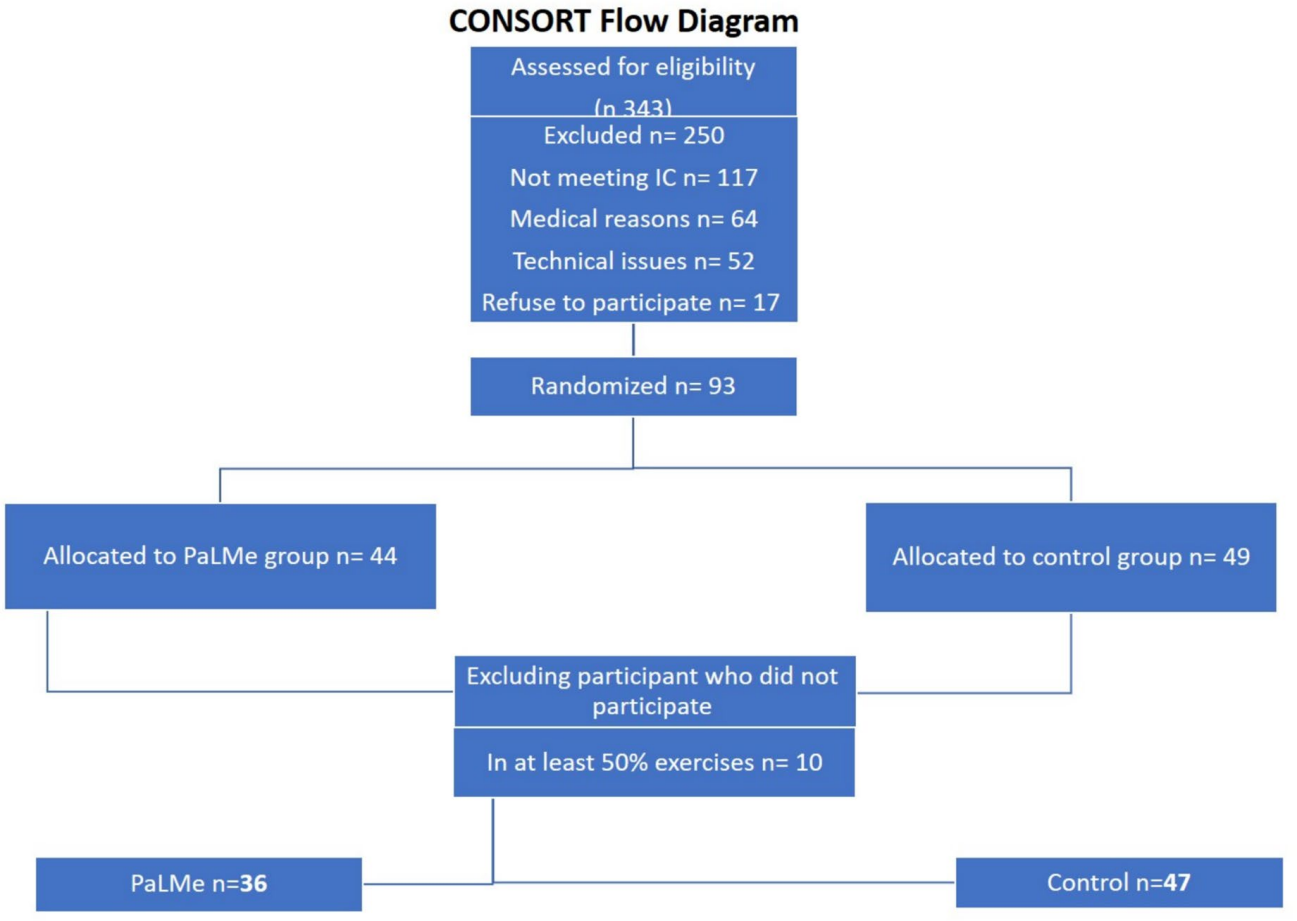
CONSORT flow diagram: participant recruitment, allocation, and analysis (IC = inclusion criteria)

Of the 93 participants enrolled, 44 were allocated to the PaLMe group and 49 to the control group. Following the predefined threshold of attending at least 50% of the prescribed exercise sessions, 11 participants were excluded from the final analysis. Consequently, data from 83 participants were analyzed: 36 in the PaLMe group and 47 in the control group. Figure 1 provides a CONSORT flow diagram illustrating the recruitment process, group allocation, and participant follow-up.

### Baseline characteristics

The baseline characteristics of the participants were as follows: the average age was 80.5 years, with 66% of participants being women and 34% men. Nearly all participants (98%) reported having a family member as a companion during hospitalization, and 96% were independent prior to the fracture. Cognitive clarity was observed in 98% of the participants, ensuring their ability to engage in rehabilitation activities. Cognitive clarity was defined as the ability to follow verbal instructions, participate actively in rehabilitation sessions, and demonstrate appropriate orientation to time, place, and situation. This assessment was based on clinical evaluation by the attending medical team, supplemented by brief bedside cognitive screening (e.g., Mini-Cog) when necessary. We have clarified this definition in response to Reviewer #1’s comments. The median hospital stay was 5.6 days. No significant differences were observed between the intervention (PaLMe) and control groups in terms of demographic or clinical characteristics.

Statistical analyses comparing the PaLMe group (paired exercises) and the control group (individual exercises) examined task ease, cognitive abilities, and motor abilities, none of which demonstrated statistical significance. Task ease, as assessed visually, had a 95% confidence interval ranging from − 0.11 to 0.385. Cognitive improvements showed a confidence interval of − 0.148–0.54, while motor improvements had a confidence interval of − 0.167–0.472. All outcomes had *p*-values more than .05, indicating no significant differences between the groups.

### Primary outcomes

1. Walking Ability

Walking Distance

**Fig. 2:**
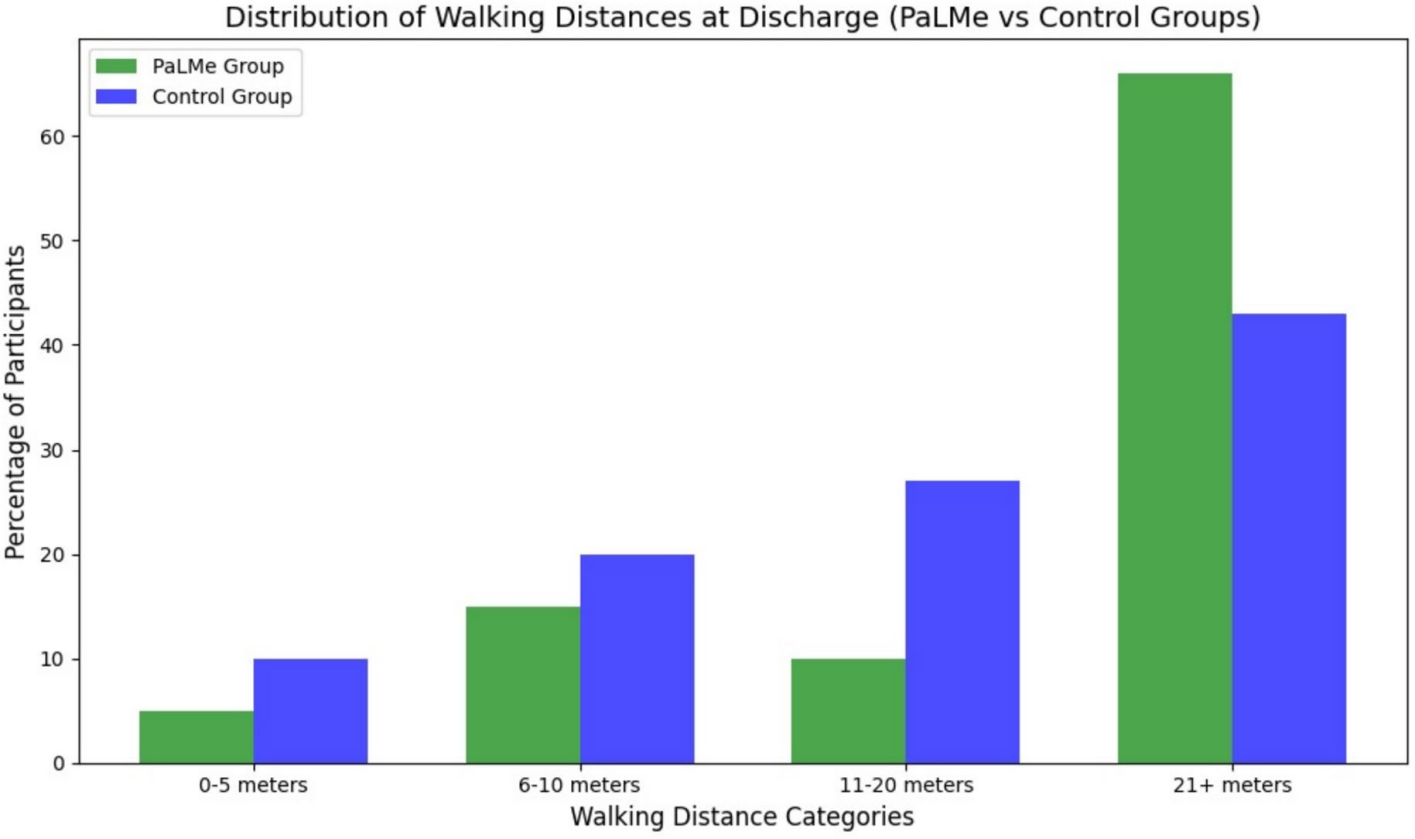
Distribution of walking distances at discharge (PaLMe vs. Control Groups)

Walking distances were recorded by physiotherapists over three consecutive days during hospitalization. Figure 2 presents a bar chart illustrating the distribution of walking distances for the PaLMe and control groups at discharge. In the PaLMe group, 66% of participants achieved walking distances of ≥ 21 m, compared to 43% in the control group (*p* < .05).

Conversely, a higher proportion of participants in the control group remained in the shorter-distance categories: 20% in the 6–10 meters category versus 15% in the PaLMe group, and 10% in the 0–5 meters category compared to only 5% in the PaLMe group. For the intermediate distance of 11–20 meters, the control group also had a higher percentage (27%) compared to the PaLMe group (10%).

Participants in the PaLMe group demonstrated greater improvements over time, with a higher proportion progressing to the longest-distance category (≥ 21 m), reflecting enhanced functional recovery. As shown in Table 1, walking distances improved significantly in the PaLMe group compared to the control group.

**Table 1:** Pre- and post-intervention outcomes for the PaLMe and control groups

Outcome	Group	Pre-intervention (Mean ± SD or N [%])	Post-intervention (Mean ± SD or N [%])	*p* value
Walking distance (m)	PaLMe	5 ± 2	21 ± 5	< 0.05
	Control	5 ± 3	15 ± 4	–
Self-efficacy (score)	PaLMe	2.5 ± 0.5	3.8 ± 0.4	< 0.05
	Control	2.6 ± 0.4	3.2 ± 0.6	–
Motivation (score)	PaLMe	3.0 ± 0.6	4.0 ± 0.5	0.02
	Control	3.1 ± 0.5	3.3 ± 0.6	–
Adherence (% completing ≥ 50% sessions)	PaLMe	–	47%	–
	Control	–	N/A	–
Complications (N [%])	PaLMe	0 (0%)	0 (0%)	–
	Control	0 (0%)	0 (0%)	–

**Fig. 3:**
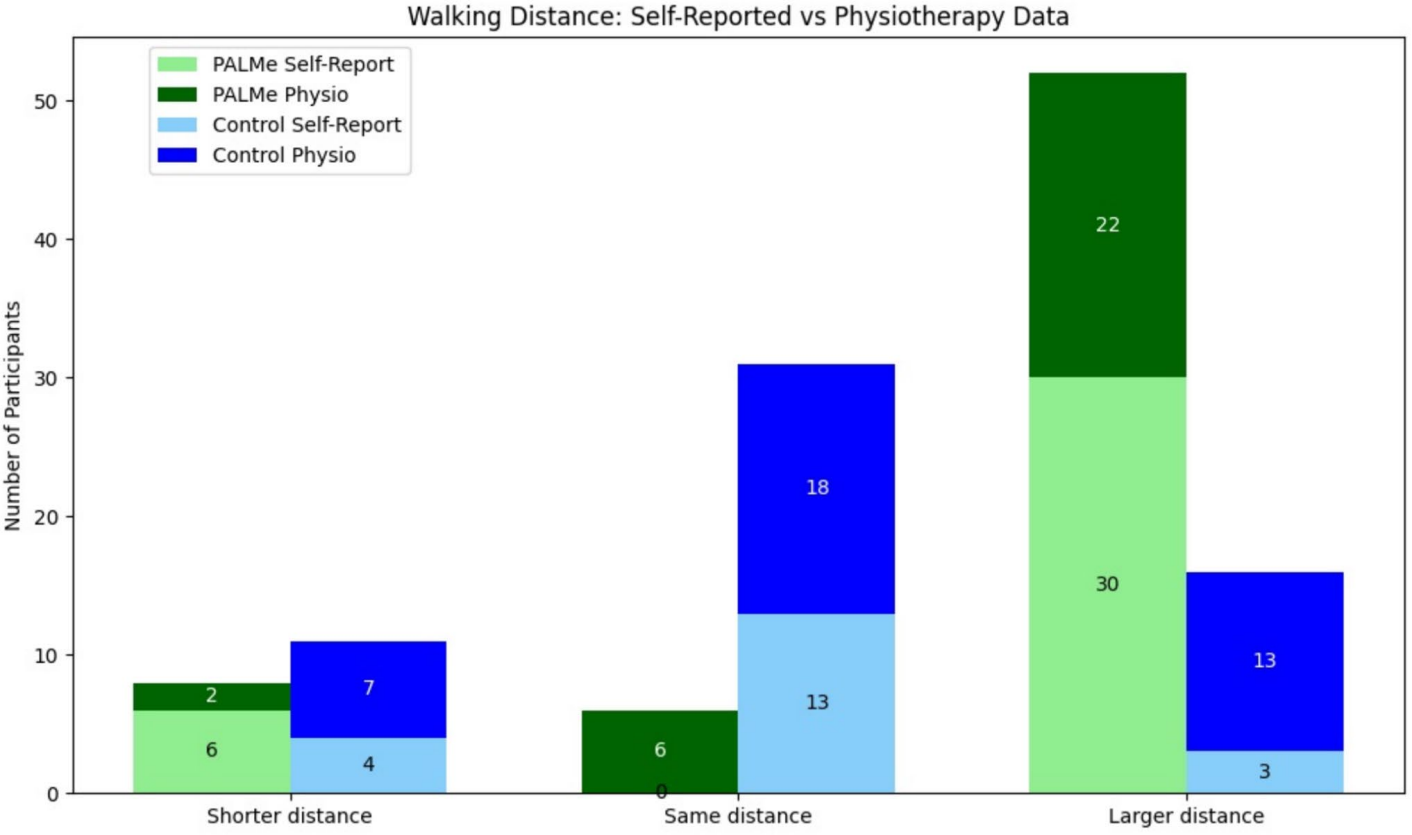
Walking distance: self-reported versus physiotherapy data. This figure compares the self-reported and physiotherapist-recorded walking distance for participants in the PaLMe intervention group and the control group at the end of hospitalization

These findings, as illustrated in Fig. 3, suggest that paired participation contributed to enhanced functional recovery, likely driven by increased motivation, peer support, and accountability (defined as the sense of responsibility participants felt toward their paired partner, encouraging adherence and engagement).

Walking Assistance

**Fig. 4:**
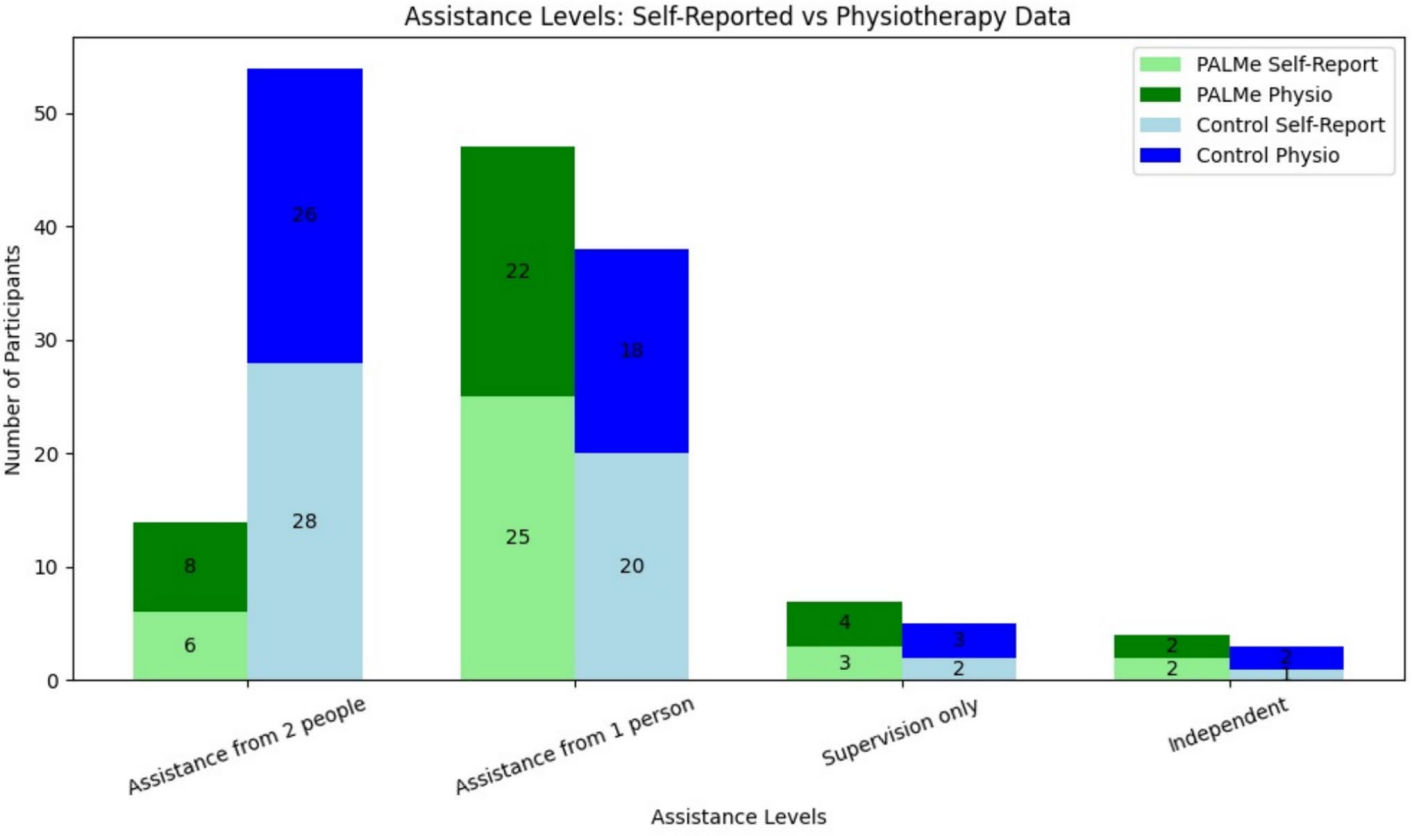
Assistance levels: self-reported versus physiotherapy data. This figure compares the self-reported and physiotherapist-recorded assistance levels for participants in the PaLMe intervention group and the control group at the end of hospitalization

The need for walking assistance was assessed based on both self-reports and physiotherapist evaluations. Assistance levels were categorized as “Independent (no assistance),” “Assistance from two people,” “Assistance from one person,” and “Supervision only (no physical assistance).” More participants in the PaLMe group achieved independence or required minimal supervision compared to the control group. These results indicate that paired exercises not only improved physical mobility but, we assume, also boosted confidence and functional independence (Fig. 4).

The median hospital stay was 5.6 days, with no significant differences observed between the PaLMe and control groups. This consistency in hospital stay duration suggests that observed differences in outcomes, such as walking ability improvement, were unlikely to be influenced by variations in recovery time in the hospital.

Feasibility

Of the 250 patients screened, 93 were recruited, resulting in a recruitment rate of 37%. The retention rate was 89%, with 83 participants completing the study. Adherence varied, with 47% of participants in the PaLMe group completing more than 50% of prescribed sessions. Participant feedback demonstrated high satisfaction with the program, with 87.7% rating the system as easy to use and 95.5% enjoying paired participation. Technical challenges were minimal, with only 10.6% of participants in the PaLMe group encountering device-related issues or scheduling conflicts. In the control group, 8.3% of participants reported device-related difficulties, though they did not experience scheduling conflicts due to the absence of a paired partner.

Complications

No cases of pressure sores or venous thrombosis were observed in either group during the study period. As a result, logistic regression analysis for these outcomes could not be performed, limiting statistical comparisons. The absence of complications is likely due to the short hospital stays and adherence to effective mobilization protocols.

### Secondary outcomes

1. Subjective Measures

PaLMe participants demonstrated significant improvements in subjective outcomes compared to the control group. A notable 62.6% of PaLMe participants rated the exercises as easy to moderate, compared to only 28.5% in the control group (*p* = .02). Overall enjoyment of the exercise program was high, with 87.7% of all participants reporting a positive experience. Among the PaLMe group, 95.5% enjoyed exercising in pairs, compared to 86.3% in the control group.

Nearly all participants felt that the exercises positively contributed to their rehabilitation, with satisfaction rates of 94% in the PaLMe group and 94.5% in the control group (*p* = NS). Notably, no technical difficulties were reported in the PaLMe group, whereas 15.1% of the control group encountered issues. This discrepancy highlights an additional benefit of the paired format, which may have facilitated better troubleshooting and peer support during the intervention.

Positive compliance with the exercise program was high across both groups, with rates of 92.8% in the PaLMe group and 98% in the control group (*p* = NS), reflecting the overall effectiveness of the rehabilitation program design (Table 2).

2. Adherence

**Table 2:** Participant feedback and compliance with the rehabilitation program: comparison between PaLMe and control groups

Variables	All participants (n = 83) (%)	PaLMe group (%)	Control group (%)	*p* Value
Enjoyed using the practice system	87.7	95.5	86.3	NS
Felt that exercises contribute	94	94	94.5	NS
Technical difficulties	10.6	0	15.1	NS
Felt the exercise was easy-moderate	45	62.6	28.5	.02
Positive compliance to exercise	95.2	92.8	98	NS

Adherence to the rehabilitation program was generally high across both groups. However, only 47% of PaLMe participants completed more than 50% of the recommended sessions, which was attributed to logistical challenges such as conflicting physiotherapy schedules, overlapping visiting hours, and limited availability of shared exercise spaces. Efforts to address these barriers, such as offering scheduling flexibility, were only partially effective.

Participants in the PaLMe group who completed a higher percentage of sessions reported finding the exercises easier, which may have supported greater engagement. Differences in perceived exercise difficulty between the groups suggest that shared participation positively influenced participants' adherence and overall experience.

### Adverse events

No adverse events related to the intervention were reported in either group. The absence of technical difficulties in the PaLMe group, compared to 15.1% in the control group, suggests that paired participation may have alleviated barriers to technology use.

## Discussion

The findings of this study demonstrate the feasibility of an online rehabilitation program for senior patients recovering from hip fracture surgery and highlight the potential of paired exercises to significantly enhance rehabilitation outcomes, particularly in terms of functional and subjective measures. This aligns with the growing interest in eHealth interventions for geriatric rehabilitation, as highlighted by Kraaijkamp et al. who reviewed the effectiveness and feasibility of such approaches [37]. However, their review did not include online programs specifically tailored for old patients recovering from hip fracture surgery, underscoring the novelty of the PaLMe program in addressing this gap. Walking distance improvements were a key outcome of the PaLMe program. At discharge, 66% of participants in the PaLMe group achieved walking distances of ≥ 21 m, compared to 43% in the control group (*p* < .05). The consistent median hospital stay of 5.6 days between the intervention and control groups reduces the likelihood that the observed differences in outcomes were influenced by longer recovery times for one group. However, while discharge timing was generally comparable, future studies should consider standardizing assessments relative to the time of surgery to further minimize variability in recovery metrics. This significant improvement highlights the potential of paired participation to enhance mobility and independence beyond standard rehabilitation. These findings align with prior studies supporting the efficacy of structured rehabilitation in older people to improve functional outcomes [37–39].

The PaLMe program demonstrated both psychological and functional benefits. Participants in the intervention group reported increased confidence and motivation, reflecting the value of shared interaction in overcoming barriers to rehabilitation. Qualitative feedback highlighted the importance of peer support, with participants describing feelings of independence, enhanced confidence, and greater motivation when exercising with a partner. Additionally, Additionally, accountability—defined as the perceived responsibility toward one’s paired partner—emerged as a key factor in sustaining engagement. Prior research suggests that peer-assisted rehabilitation models improve adherence by fostering a sense of shared responsibility, reducing dropout rates, and enhancing long-term compliance with physical therapy [28, 29]. This aligns with our participants’ feedback, where several individuals reported feeling more committed to completing their exercises knowing that their partner was also involved. 62.6% of PaLMe participants rated the exercises as easy to moderate compared to only 28.5% in the control group (*p* = .02), suggesting that shared accountability reduced the perceived difficulty of rehabilitation exercises. These findings align with research demonstrating that social interaction reduces depression and anxiety in older adults, fostering emotional well-being and adherence to rehabilitation programs, as well as prior studies highlighting the efficacy of structured rehabilitation in aging populations [40–44].

No cases of pressure sores or venous thrombosis were observed in either group during the study period. This outcome likely reflects the short hospital stays and adherence to effective mobilization protocols, which are known to mitigate these complications. However, the absence of these complications limited the logistic regression analysis, making it impossible to calculate odds ratios or identify significant differences.

Evaluating feasibility was a primary aim of this study, and the results demonstrate that the PaLMe program is both acceptable and practical for older patients recovering from hip fracture surgery. The recruitment and retention rates (37% and 89%, respectively) highlight the program’s appeal and participant engagement, while positive feedback on usability and paired participation underscores its potential to address psychological and motivational barriers to rehabilitation. However, logistical challenges, including scheduling conflicts, visitor appointments, and limited availability of shared exercise spaces, impacted adherence, with only 47% of participants in the PaLMe group completing more than half of the prescribed sessions. Despite these barriers, compliance with the rehabilitation program was strong in both groups (PaLMe: 92.8%; control: 98%). Addressing logistical issues, such as by optimizing scheduling flexibility, dedicating time slots, and increasing staff resources, could further enhance the feasibility and scalability of the PaLMe program, supporting its integration into broader clinical settings.

Conflicting treatment schedules, visitor appointments, and limited availability of shared exercise spaces posed logistical challenges to the implementation of the PaLMe program [45]. While scheduling flexibility was attempted, its partial effectiveness highlights the need for innovative strategies, such as dedicated time slots, additional staff resources, and optimized schedules, to improve adherence. Despite these challenges, compliance with the rehabilitation program was strong in both groups (PaLMe: 92.8%; control: 98%). However, the sub-optimal participation rate in the PaLMe group (47% completing more than 50% of the prescribed exercises) underscores the importance of addressing these logistical barriers to maximize the program’s potential.

Qualitative feedback further highlighted the psychological and social benefits of paired participation. Participants described feeling more independent, confident, and motivated when exercising with a partner, suggesting that the emotional support provided by peer interaction significantly contributes to recovery [46]. These findings align with research showing that social interaction reduces depression and anxiety in older adults, fostering emotional well-being, adherence, and improved physical outcomes [47–50]. The PaLMe program demonstrates that pairing patients can provide a low-cost, scalable intervention with benefits extending beyond physical recovery.

Despite these promising findings, the study faces several limitations. The small sample size of the intervention group (36 participants) limits the generalizability of the results. The power calculation, based on a dichotomous endpoint, may also have limitations given the low incidence of the primary outcome, which could affect the robustness of the findings. Additionally, walking distance, a continuous variable, was categorized into clinically meaningful thresholds to improve interpretation and comparability with prior studies. This approach reduces the impact of extreme values, aligns with clinical rehabilitation goals, and ensures the findings are actionable for patient management. However, future studies with larger sample sizes could explore analyzing walking distance as a continuous variable to provide additional granularity in recovery trajectories.

Furthermore, we conducted a per-protocol analysis, focusing on participants who fully adhered to the intervention. While this approach allows for a clearer assessment of the intervention’s efficacy in compliant individuals, it does not reflect an intention-to-treat analysis, which would have provided a more conservative estimate by including all randomized participants, regardless of adherence. Future studies should consider incorporating an ITT (Intension to treat) Cognitive clarity was observed in 98% of the participants, analysis to enhance generalizability and account for real-world adherence variability.

Additionally, the short study period does not allow for the evaluation of long-term outcomes, such as sustained mobility improvements or reductions in chronic complications. While this study was conducted in a controlled hospital setting, real-world rehabilitation involves challenges such as unpredictable surgical delays, varying patient admission patterns, and resource availability fluctuations. To better understand the intervention’s real-world applicability, future research should assess its feasibility in standard rehabilitation settings where these constraints exist. Additionally, examining long-term adherence and patient satisfaction in outpatient or home-based rehabilitation settings could further inform its clinical utility. Furthermore, the exclusion of patients whose surgery was delayed beyond 48 h introduces a selection bias. This exclusion was necessary to reduce variability in postoperative recovery, as delayed surgery has been associated with poorer functional outcomes. However, this criterion limits the applicability of our findings to patients experiencing surgical delays. Future research should investigate whether the intervention remains effective in this subgroup. Another limitation is the exclusion of patients admitted on Thursdays or Fridays due to reduced rehabilitation staff availability on weekends. This decision was made to ensure standardization of therapy initiation, as early mobilization is a key factor in recovery. However, this exclusion limits the generalizability of our findings to all hospitalized patients. Future studies should examine the feasibility of implementing the PaLMe program across all admission days to assess its applicability in routine clinical practice. Although the intervention demonstrated significant improvements, the relatively small number of analyzed participants remains a limitation. While our findings suggest meaningful clinical benefits, larger multicenter trials are needed to confirm these effects in a broader population and assess long-term outcomes. Finally, while this study was conducted in a controlled hospital setting with strict inclusion criteria, real-world rehabilitation involves variability in patient admission timing, surgical delays, and resource availability. To enhance external validity, future research should assess the feasibility and effectiveness of paired rehabilitation under standard clinical conditions, including diverse patient pathways and potential logistical constraints. Artificial intelligence (AI) is increasingly influencing clinical decision-making and rehabilitation strategies. While AI models can predict recovery patterns and optimize treatment plans, they cannot replace well-designed clinical trials that evaluate patient engagement and adherence to interventions. Future research may explore AI-assisted patient pairing algorithms to optimize rehabilitation experiences while maintaining the evidence-based framework of structured clinical trials.

## Conclusions

The PaLMe program demonstrates a feasible, effective approach to rehabilitation post-hip fracture surgery, enhancing mobility, self-efficacy, and motivation. Addressing logistical barriers could further improve adherence and outcomes, paving the way for broader implementation.

## Data Availability

The data that support the findings of this study are available from the corresponding author upon reasonable request. Due to ethical and privacy considerations, the data are not publicly available.
